# *p15^INK4b^* in bladder carcinomas: decreased expression in superficial tumours

**DOI:** 10.1054/bjoc.2001.2106

**Published:** 2001-11

**Authors:** M A Le Frère-Belda, D Cappellen, A Daher, S Gil-Diez-de-Medina, F Besse, C C Abbou, J P Thiery, E S Zafrani, D K Chopin, F Radvanyi

**Affiliations:** Service d'Anatomie et de Cytologie Pathologiques, Oncogénèse respiratoire et uro-génitale EMI 99-09 et Service d'Urologie, Centre Hospitalier Universitaire Henri Mondor, Créteil Cedex, 94010, France; UMR 144, CNRS – Institut Curie, Section de Recherche, 26 rue d'Ulm, Paris Cedex 05, 75248, France

**Keywords:** bladder, human transitional cell carcinoma, cyclin-dependent kinase inhibitor, *p15*

## Abstract

The *p15* gene which encodes a cyclin-dependent kinase inhibitor, is located in the 9p21 chromosomal region that is frequently deleted in human bladder transitional cell carcinomas (TCCs). The aim of the present paper is to study the potential involvement of the *p15* gene in the evolution of TCCs. *p15* mRNA expression was investigated by semi-quantitative RT-PCR in a series of 75 TCCs, 13 bladder cell lines and 6 normal bladder urothelia by semi-quantitative RT-PCR. *p15* was expressed in the normal urothelium but *p15* mRNA levels were significantly decreased in 66% of the superficial (Ta-T1) TCCs (*P* = 0.0015). In contrast, in muscle-invasive (T2-T4) TCCs, *p15* expression differed widely between samples. *p16* mRNA levels were also studied and there was no correlation between *p15* and *p16* mRNA levels, thus indicating that the two genes were regulated independently. Lower *p15* expression in superficial tumours did not reflect a switch from quiescence to proliferative activity as normal proliferative urothelial controls did not present decreased *p15* mRNA levels relative to quiescent normal urothelia. We further investigated the mechanisms underlying *p15* down regulation. Homozygous deletions of the *p15* gene, also involving the contiguous *p16* gene, were observed in 42% of the TCCs with decreased *p15* expression. No hypermethylation at multiple methylation-sensitive restriction sites in the 5́-CpG island of *p15* was encountered in the remaining tumours. Our data suggest that decreased expression of *p15* may be an important step in early neoplastic transformation of the urothelium and that a mechanism other than homozygous deletions or hypermethylation, may be involved in *p15* down regulation.© 2001 Cancer Research Campaign  http://www.bjcancer.com


British Journal of Cancer (2001) 85(10), 1515–1521
© 2001 Cancer Research Campaign
doi: 10.1054/ bjoc.2001.2106, available online at http://www.idealibrary.com on IDEAL

### *p15*[INK4b] in bladder carcinomas: decreased expression in superficial tumours

**MA Le Frère-Belda[1,2], D Cappellen[3], A Daher[2], S Gil-Diez-de-Medina[2,3], F Besse[3], CC Abbou[2], JP Thiery[3], ES Zafrani[1], DK Chopin[2,4] and F Radvanyi[3]**

[1]Service d'Anatomie et de Cytologie Pathologiques; [2]Oncogénèse respiratoire et uro-génitale EMI 99-09 et Service d'Urologie, Centre Hospitalier Universitaire Henri Mondor, 94010 Créteil Cedex, France and [3]UMR 144, CNRS – Institut Curie, Section de Recherche, 26 rue d'Ulm, 75248 Paris Cedex 05, France

**Summary** The *p15* gene which encodes a cyclin-dependent kinase inhibitor, is located in the 9p21 chromosomal region that is frequently deleted in human bladder transitional cell carcinomas (TCCs). The aim of the present paper is to study the potential involvement of the *p15* gene in the evolution of TCCs. *p15* mRNA expression was investigated by semi-quantitative RT-PCR in a series of 75 TCCs, 13 bladder cell lines and 6 normal bladder urothelia by semi-quantitative RT-PCR. *p15* was expressed in the normal urothelium but *p15* mRNA levels were significantly decreased in 66% of the superficial (Ta-T1) TCCs ($P = 0.0015$). In contrast, in muscle-invasive (T2-T4) TCCs, *p15* expression differed widely between samples. *p16* mRNA levels were also studied and there was no correlation between *p15* and *p16* mRNA levels, thus indicating that the two genes were regulated independently. Lower *p15* expression in superficial tumours did not reflect a switch from quiescence to proliferative activity as normal proliferative urothelial controls did not present decreased *p15* mRNA levels relative to quiescent normal urothelia. We further investigated the mechanisms underlying *p15* down regulation. Homozygous deletions of the *p15* gene, also involving the contiguous *p16* gene, were observed in 42% of the TCCs with decreased *p15* expression. No hypermethylation at multiple methylation-sensitive restriction sites in the 5´-CpG island of *p15* was encountered in the remaining tumours. Our data suggest that decreased expression of *p15* may be an important step in early neoplastic transformation of the urothelium and that a mechanism other than homozygous deletions or hypermethylation, may be involved in *p15* down regulation. © 2001 Cancer Research Campaign http://www.bjcancer.com

**Keywords**: bladder; human transitional cell carcinoma; cyclin-dependent kinase inhibitor; *p15*

In eukaryotes, cell cycle progression is particularly controlled at two steps, before the transitions from G1 to S and from G2 to M (Hall and Peters, 1996). Progression through both checkpoints is controlled by cyclin-dependent protein kinases (CDKs) sequentially regulated by cyclins D, E and A (Sherr, 1996). P15 (also called p15$^{INK4b}$, MTS2, INK4b, CDKN2B) and p16 (also known as p16$^{INK4a}$, p16$^{INK4}$, MTS1, CDK4I, CDKN2A) prevent CDK activation, specifically that of CDK4 and CDK6 associated with D-type cyclins, by blocking the binding to the cyclin regulatory subunits, inducing G1 phase arrest (Hannon and Beach, 1994; Serrano et al, 1993). However, the activities of p15 and p16 are regulated differently. P15 is an effector of transforming growth factor-β (TGF-β)–induced cell cycle arrest whereas p16 is not involved in TGF–β–induced growth inhibition (Hannon and Beach, 1994). *P15* is located on chromosome band 9p21 adjacent to the *INK4a/ARF* locus, which encodes two unrelated proteins, p16$^{INK4a}$ and p14$^{ARF}$, through the use of shared coding regions and alternative reading frames. P14$^{ARF}$ is a potent negative regulator of the cell cycle that functions in a manner different from that of CDK inhibitors, via a p53-dependent pathway (Sherr, 1998; Sharpless and DePinho, 1999). The *INK4a/ARF* locus is a frequent site of chromosomal deletion in human tumours (Hannon and Beach, 1994; Jen et al, 1994). Numerous studies have identified *p16* as the principal target of these deletions (Kamb, 1995). Mutational analysis has shown that *p16* is commonly mutated or homozygously deleted in human cancer. In particular, germline mutations specifically affecting *p16* have been identified in familial melanoma (Hall and Peters, 1996; Sherr, 1996). The methylation of the 5'-CpG island of *p16* has been proposed as another mechanism for the inactivation of this gene (Merlo et al, 1995; Herman et al, 1995). The neighbouring *p15* gene has been considered as a putative tumour suppressor gene due to the high level of sequence identity and functional similarity between p15 and p16. The analysis of cell lines and primary tumours of various origins has not resulted in the identification of any *p15* gene point mutations (Stone et al, 1995; Hall and Peters, 1996; Sherr, 1996). Homozygous deletions of the *p15* gene in primary tumours and tumour cell lines almost invariably involves the nearby *p16* gene as well. Aberrant methylation of *p15* is associated with the loss of transcription of this gene in leukaemias and gliomas (Herman et al, 1996). This mechanism, which seems to involve the *p15* gene selectively, provides the sole evidence so far of a tumour suppressor role for this gene in human neoplasia.

Urinary bladder transitional cell carcinomas (TCCs) is the fourth most common cancer in men and the ninth most common cancer in women in Western countries. TCCs are either superficial [Ta-T1 tumours including TCCs confined to the urothelium (Ta) and those invading only the lamina propria (T1)]or muscle-invasive. The 9p21 region, surrounding the *INK4a/ARF* locus and the *p15* gene, has been found to be lost in about 50% of bladder tumours (Reznikoff et al, 1996). Several analyses of bladder tumours have failed to identify *p15* gene point mutations (Orlow et al, 1995; Packenham et al, 1995; Miyamoto et al, 1995). Although the homozygous deletions found in bladder tumours generally

*Received 24 January 2001*
*Revised 30 July 2001*
*Accepted 7 August 2001*

*Correspondence to:* DK Chopin include both *p15* and *p16*, rare examples of selective *p15* deletion have been described (Orlow et al, 1995; Packenham et al, 1995; Williamson et al, 1995). No study has reported the inactivation of *p15* through a loss of expression. Basing our study on the clear involvement of the loss of the 9p21 region in bladder cancer, we evaluated the importance of the cell cycle inhibitor *p15* in bladder carcinogenesis, by investigating changes in *p15* expression at the mRNA level in a series of 75 primary TCCs of various stages and grades and 13 bladder cancer cell lines. As we found a decreased expression of *p15* in a significant number of bladder tumours, we investigated several potential mechanisms for this down regulation. Considering the known involvement of *p16* in some tumours, we have also analysed *p15* expression in conjunction with *p16* expression.

### MATERIALS AND METHODS

### Cell lines

Human bladder cell lines 647V, EJ138, J82, JON53, RT112, T24, TCCSUP were obtained and cultured as previously described (Gil Diez de Medina et al, 1999). Lysates in 4M guanidinium thiocyanate of the bladder cell lines HCV29, HT1376, RT4, UM-UC-3, VM-CUB-1, VM-CUB-3, were kindly provided by Dr J Southgate (Leeds, U.K.).

### Tissue samples

Tumour tissues were obtained from transurethral resections or radical cystectomy samples from 75 patients with transitional cell carcinomas of the urinary bladder. Tumours were classified by stage according to the TNM classification (UICC, 1992) and by grade according to criteria recommended by the World Health Organisation (WHO, 1973). The tumours studied were: 9 Ta (papillary superficial non-invasive tumours), 14 T1a (lesions invading the superficial lamina propria), 15 T1b (lesions invading the deep lamina propria), 10 T2 (lesions invading the inner layer of vesical muscle), 17 T3 (outer layer of the muscle invaded with or without adipous perivesical tissue tumour invasion), and 10 T4 (tumour extension beyond the bladder). Sixteen were grade G1 (low grade), 26 grade G2 (intermediate grade), and 33 grade G3 (high grade). A representative sample was taken from each tumour for histological assessment, and an adjacent fragment was placed in liquid nitrogen and stored at –80˚C for subsequent DNA and RNA extraction. Each sample included in this analysis contained more than 80% malignant cells, as assessed by histological examination. Fourteen normal samples obtained during organ procurement for transplantation on cadaveric donors were also studied. They consisted of six urothelium samples obtained by scraping off the urothelium from the submucosa of normal bladder specimens, three lamina propria samples and five vesical smooth muscle specimens, dissected separately.

### Primary cultures of human urothelia and 5-bromo-2´-deoxyuridine (BrdU) incorporation

An organo-typic culture model (de Boer et al, 1996) was used for three normal urothelia. In order to compare *p15* mRNA expression to proliferation data in these primary cultures of human normal urothelia, BrdU incorporation was studied at different times of the culture (de Boer et al, 1994). Briefly, upon termination of the culture, cells were incubated with 40 µg/ml of BrdU in serum-free medium for 2 h. Cultures were then rinsed with PBS, PH 7.2, and fixed with 96% ethanol for at least 1 h for immunocytochemistry. Before the primary anti-BrdU antibody incubation, cultures were treated with HCl and Borax buffer. Chain-specific cytokeratin and BrdU expressions were visualized using appropriate dilutions of the primary mouse monoclonal antibodies in a conjugated immunoenzyme assay. The anti-cytokeratin and anti-BrdU antibodies were kindly donated by Professor FCS Ramaekers (Maastricht, Netherlands). Secondary rabbit anti-mouse (Dako, Glostrup, Denmark) antibodies were either peroxidase-conjugated (used for BrdU staining) or alkaline phosphatase-conjugated (used for cytokeratin staining). 3,3´-diaminobenzidine tetrahydrochloride (DAB) or the diazonium salt served as chromogens, and fast red violet LB with naphtol AS-MX phosphate served as coupling reagent (Sigma, St Louis, USA). The number of cells immunostained with the anti-BrdU antibody was then compared to the total cell number assessed with the anti-cytokeratin antibody in 1 $mm^2$ surface.

### RNA and DNA extraction

RNA and DNA were extracted simultaneously using the caesium chloride cushion method essentially as described elsewhere (Coombs et al, 1990), but with slight modifications (Cappellen et al, 1997; Gil Diez de Medina et al, 1997).

### RT-PCR

*p15* and *p16* messenger RNA levels were determined by radioactive semi-quantitative RT-PCR using TBP (TATA-binding protein) or GAPDH as internal controls. cDNA synthesis and PCR analysis were performed as previously described (Radvanyi et al, 1993; Gil Diez de Medina et al, 1997). Briefly, the number of cycles was chosen to be in the exponential part of the PCR (23 cycles for the co-amplification of p15 and TBP, 24 for the co-amplification of p16 and TBP and 21 for the co-amplification of TBP and GAPDH). The primer sequences used for p15 and p16, located in exons 1 and 2 of these genes, were: 5´-CGCTGCCCATCATCATGAC-3´ (sense) and 5´-CTAGTGGAGAAGGTGCGACA-3´ (antisense) for p15 and 5´-CCAACGCACCGAATAGTTAC-3´ (sense) and 5´-CACGGGTCGGGTGAGAGT-3´ (antisense) for p16. Primer sequences for TBP and GAPDH were as described elsewhere (Gil Diez de Medina et al, 1997). The PCR-amplified products were subjected to electrophoresis in 8% polyacrylamide gels. Signals were quantified with a Molecular Dynamics 300 PhosphorImager (Molecular Dynamics, Sunnyvale, CA). There was no amplification if reverse transcriptase was omitted from the reverse transcription reaction.

### Homozygous deletions analysis for the p15 and p16 genes

Homozygous deletions were detected by a PCR based assay. A fragment located in exon 1 of *p15* (or *p16*) was co-amplified with a genomic fragment of either *GAPDH* or *PSA* as a control. *GAPDH* and *PSA* are located in chromosomal regions that are infrequently the target of allelic loss in bladder carcinomas (less than 3% and 7%, respectively). The primer sequences for *p15*, *p16*, *GAPDH* and *PSA* are listed below.

5´-GGCCAGAGCGGCTTTGAG-3´ (*p15* sense) and 5´-CTGG GCTCAGCTTCATTACC-3´ (*p15* antisense), 5´-TCGGGTAGAGGAGGTGCGGG-3´ (*p16* sense) and 5´-GATCGGCCTCCGACCGTAACT-3´ (*p16* antisense), 5´-TGGGGTGGTGAATA

 *© 2001 Cancer Research Campaign*

CC ATGT-3´ (*GAPDH* sense) and 5´-AAGGCATGGCTGCAACT GAA-3´ (*GAPDH* antisense), 5´-AGGCTGGGGCAGCAT-3´ (*PSA* sense) and 5´-CACCTTCTGAGGGTGAACTTG-3´ (*PSA* antisense). PCR were performed with 50 ng of genomic DNA and the number of cycles was selected so as to be in the exponential part of the two amplification reactions (i.e. 23 cycles). PCR products were analysed as described in the reverse transcription-PCR section. The relative intensity of the products obtained for the test (*p15* or *p16*) and control (*GAPDH* or *PSA*) sequences in normal and tumour DNA samples was compared and the relative representation of *p15* and *p16* calculated as follows:

$$\frac{\text{Intensity of test sequence in tumour DNA}}{\text{Intensity of control in tumour DNA}} \times \frac{\text{Intensity of control in normal DNA}}{\text{Intensity of test sequence in normal DNA}}$$

Given the potential for tumour heterogeneity and contamination with non-neoplastic cells, tumours with ratios below 0.3 were considered to have homozygous deletions.

### Methylation analysis

A quantitative PCR assay based on the inability of some restriction enzymes to cut methylated sequences (Singer-Sam et al, 1990) was used to analyse the methylation status of the first exon of the *p15* gene. Three sets of primers flanking three different regions of exon 1 of *p15* gene were designed (Figure 3). The sites examined were: one *Hpa*II site in fragment 1, one *Eag*I, two *Hpa*II and five *Cfo*I sites in fragment 2 and two *Hpa*II, one *Sac*II and ten *Cfo*I sites in fragment 3. DNA was digested according to the manufacturer's instructions (New England Biolabs). DNA (1 µg) was digested overnight at 37˚C, with 10 units of enzyme/µg of DNA. The primer sets used for methylation analysis of *p15* exon 1 were 5´-CCTTGGCCCAGCT-GAAAACG-3´ (sense) and 5´-ACGCAGCCGAGCTCAAAGC-3´ (antisense) for fragment 1 and, 5´-CGGCCAACGGTGGAT-TATCC-3´ (sense) and 5´-CACACCTCGCCAACGTAGAC-3´ (antisense) for fragment 3. The primer set for fragment 2 and the amplification reactions were as described in the homozygous deletions analysis section except that 25 and 26 cycles were performed to amplify fragments 1 and 3 of exon 1 of the *p15* gene respectively, so as to be in the linear range of the assay. The PCR-amplified products were subjected to electrophoresis in 8% polyacrylamide gels and an autoradiograph was produced. *Afl*II restriction enzyme was used as a positive control (restriction site outside the amplified fragments) and *Msp*I restriction enzyme as a negative control (methylation-insensitive enzyme) for each template.

### Statistical analysis mRNA levels were analysed according to stage and frequencies were analysed with the Mann-Whitney test. Correlation was estimated between *p15* and *p16* mRNA expression.

### RESULTS

### Decreased expression of *p15* mRNA in superficial TCCs

*p15* mRNA levels were determined in normal bladder tissues (urothelium, lamina propria, muscle) and in a series of 75 TCCs (38 superficial and 37 muscle-invasive tumours) by semi-quantitative RT-PCR, using two different internal controls, TBP and GAPDH (Figures 1, 2A and data not shown). The six normal human urothelia studied all expressed *p15* mRNA, and the levels in the various urothelia were similar (mean value = 0.86). *p15* mRNA was also detected in lamina propria ($n$ = 3) and muscle ($n$ = 5) (mean value 0.51 and 0.53 respectively). The superficial TCCs (Ta, T1a, T1b) had significantly lower levels of *p15* mRNA than normal urothelium ($P$ = 0.0015). Twenty-five of the 38 superficial TCCs (66%) contained low levels of *p15* mRNA (less than 30% of the mean value for normal urothelium) and in 19 of these 25 TCCs, *p15* mRNA levels were close to zero. Such a decrease in *p15* mRNA levels was more frequent in the Ta-T1a tumours. In this group, 18 out of 23 tumours (78%) presented low levels of expression, versus 7 out of 15 T1b tumours (47%). In the superficial tumours that expressed *p15*, *p15* mRNA levels were similar to those for normal urothelium. In contrast, in the 37 invasive TCCs (T2–T4), *p15* mRNA levels varied widely from non-detectable to more than 5 times higher than the level found in normal urothelium and no significant difference in *p15* mRNA level was found between normal urothelium and invasive TCCs ($P$ = 0.55). We compared

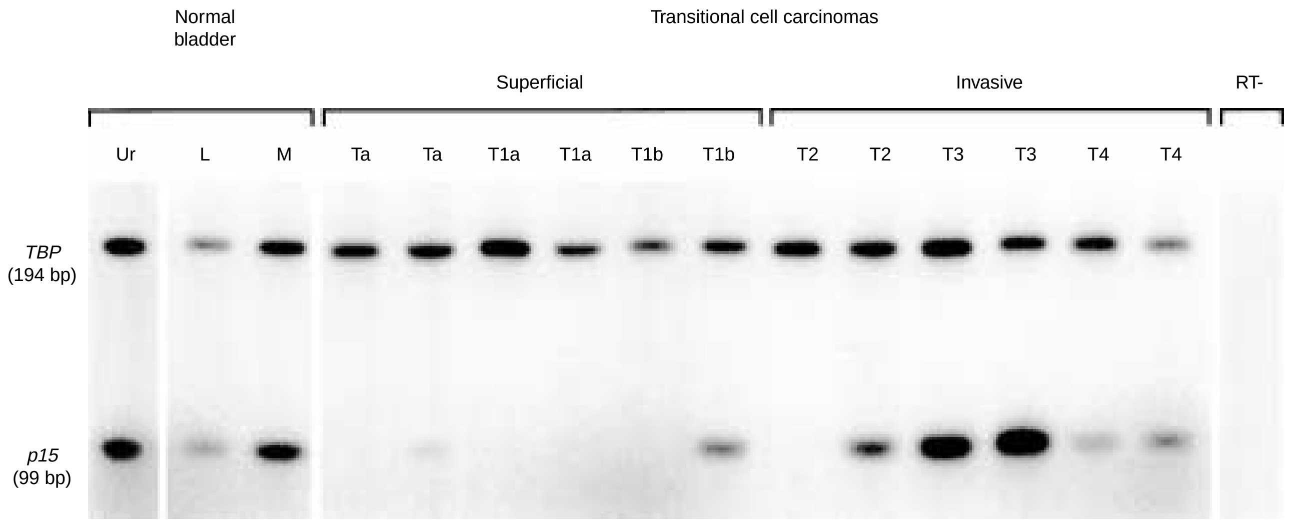

**Figure 1** *p15* mRNA levels in normal bladder (Ur, urothelium; L, lamina propria; M, smooth muscle) and primary bladder carcinomas of various stages, determined by semi-quantitative RT-PCR, using TBP as an internal standard. RT-, PCR performed on an RNA sample from normal urothelium, incubated in reverse transcription buffer without reverse transcriptase. Examples of *p15* levels in normal tissues and in TCCs of various stages are shown

*© 2001 Cancer Research Campaign*

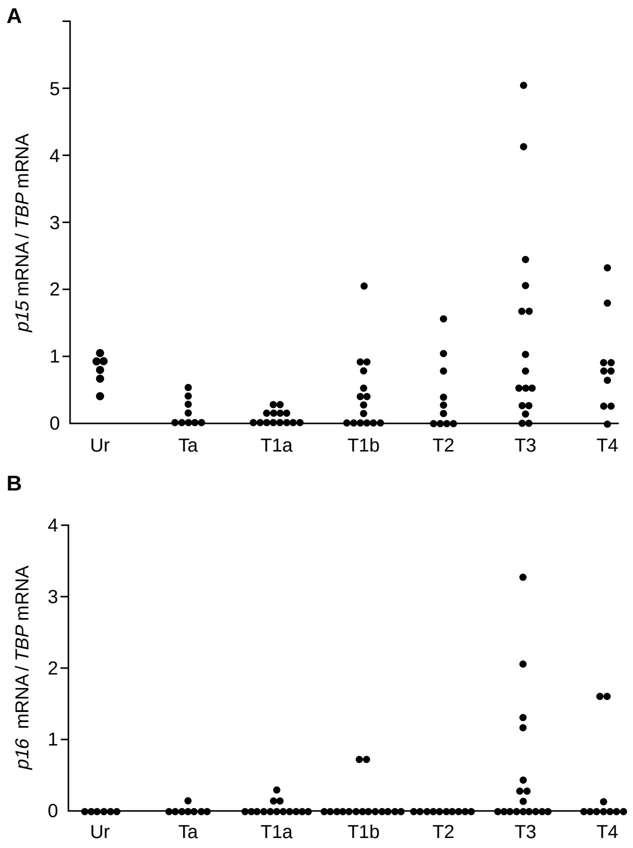

**Figure 2** Semi-quantification data (see Materials and Methods) of *p15* mRNA/*TBP* mRNA (**A**) and *p16* mRNA/*TBP* mRNA (**B**) in urothelium (Ur) and TCCs according to stage. *p15* mRNA was expressed in normal urothelium and significantly decreased in superficial tumours (mainly Ta-T1a). In muscle invasive-TCCs (T2–T4), *p15* expression differed widely between samples. On the other hand, *p16* mRNA was not expressed in normal urothelium. In superficial TCCs, *p16* mRNA was in most cases low or non-detectable whereas in invasive TCCs, *p16* mRNA levels were various. No correlation was found between *p15* mRNA and *p16* mRNA levels (correlation coefficient $R^2$ = 0.26)

*p15* mRNA levels with tumour grade and found that 12 out of 16 G1 (75%), 13 out of 26 G2 (50%) and 9 out of 33 G3 (27%) had *p15* mRNA levels less than 30% of the mean for normal urothelium. Similar results were obtained if *GAPDH* was used as the internal standard instead of *TBP* (data not shown).

### *p15* mRNA expression in proliferative normal human urothelia in an organo-typic culture model

Cell proliferation on the cultures of normal urothelia was assessed by BrdU incorporation. The highest level of proliferation was obtained after 5 days' culture (19.4% of cells immunostained with the anti-BrdU antibody) and then it decreased slowly after 10 days' culture. The analysis of *p15* mRNA levels in this model showed that *p15* mRNA expression, present in quiescent urothelia, did not decrease in proliferative urothelia (Table 1).

### Absence of *p16* mRNA expression in normal bladder tissues, in most superficial TCCs and in some invasive TCCs

*p16* mRNA expression was studied in all the samples of normal bladder tissues and in 74 (37 superficial and 37 muscle invasive tumours) out of the 75 TCCs previously studied for *p15* mRNA

**Table 1** Quantitation of paramaters for proliferation and *p15* mRNA expression in six normal quiescent urothelia and in three primary cultures of human urothelia. *p15* mRNA expression/*TBP* mRNA is the mean of the ratio of *p15* mRNA to *TBP* mRNA in semi-quantitative RT-PCR ± the standard deviation. The proliferation was determined as described in Materials and Methods and is given as the mean of the percentage of BrdU positive nuclei relative to the total number of nuclei ± the standard deviation

| | Day of culture | |
|---|---|---|
| | **D0** | **D10** |
| *p15* mRNA/TBP mRNA | 0.9 ± 0.2 | 4.9 ± 2.7 |
| BrdU incorporation | 3 ± 0.5 | 19.4 ± 3.4 |

expression (Figure 2B). *p16* mRNA levels were non-detectable in any of the normal bladder tissues whether they were urothelium, lamina propria or muscle samples. In the same way, *p16* mRNA levels were non-detectable in most of the superficial TCCs; only 6 superficial tumours (1 Ta, 3 T1a and 2 T1b) out of the 37 (16%) expressed *p16*. On the other hand, *p16* positive tumours were more frequent in the invasive TCCs group and concerned 13 (2 T2, 8 T3, 3 T4) of the 37 tumours (35%). No correlation was found between *p15* and *p16* mRNA expression in the TCCs (correlation coefficient $R^2$ = 0.26). *p16* mRNA expression was also studied according to tumour grade. *p16* mRNA levels were non-detectable in all the 15 G1 tumours as well as in 20 of the 26 G2 (77%) and 20 of the 33 G3 (61%) tumours.

### Homozygous deletions of *p15* in bladder tumours

The results are summarized in Table 2. Twenty-eight TCCs for which DNA was available were tested for deletions of exon 1 of *p15* using a PCR-based assay. They comprised 12 TCCs (1 Ta, 5 T1a, 2 T1b, 1 T2, 1 T3 and 2 T4) with p15 mRNA levels less than 30% of the mean value for normal urothelium and 16 TCCs (1 Ta, 2 T1a, 2 T2, 4 T3 and 7 T4) with higher levels of *p15* expression. Homozygous deletions of the *p15* gene were observed in 5 of the 12 tumours lacking *p15* mRNA. No homozygous deletions were detected in the 16 TCCs that expressed *p15*. The frequency of *p15* homozygous deletions was 18% if the entire cohort of TCCs was considered (5 out of 28) and 42% if the cohort was restricted to the 12 TCCs that did not express *p15* (5 out of 12). Homozygous deletions of *p15* were found in two (1 T1a, 1 T1b) of the eight superficial TCCs and in three of the four invasive tumours. The 12 TCCs lacking *p15* expression comprised 3 G1, 6 G2 and 3 G3 tumours and the 5 homozygous deletions of *p15* involved 3 of the 6 G2 and 2 of the 3 G3 tumours.

### Homozygous deletion of *p16* in bladder tumours

To determine whether *p15* deletions were selective or also involved the *p16* gene, the 5 TCCs with homozygous *p15* deletions were tested

**Table 2** Homozygous deletions of *p15* in bladder tumours according to *p15* mRNA expression and tumour stage. (– or + : *p15* mRNA levels below or above, respectively, 30% of the mean for normal urothelium)

| | ***p15* mRNA expression** | | | |
|---|---|---|---|---|
| | **–** | | **+** | |
| Tumour stage | Superficial (*n* = 8) | Invasive (*n* = 4) | Superficial (*n* = 3) | Invasive (*n* = 13) |
| Homozygous deletion | 2/8 | 3/4 | 0/3 | 0/13 |

 *© 2001 Cancer Research Campaign*

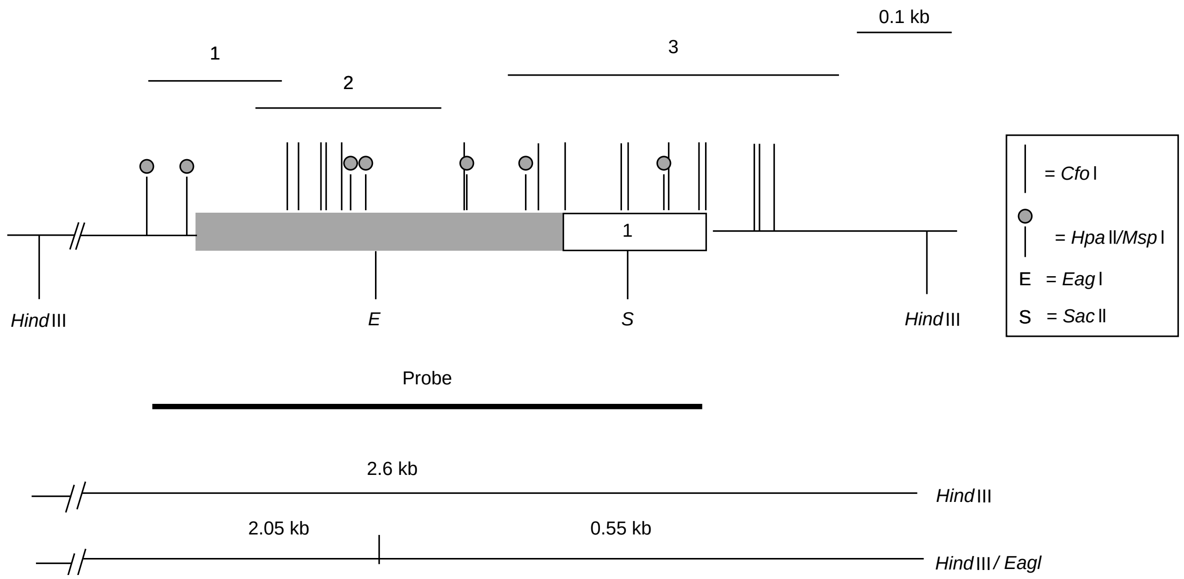

**Figure 3** Schematic map of exon 1 of the *p15* gene. Exon 1 is depicted with the non-coding region shaded grey. The positions of multiple methylation-sensitive restriction enzyme sites and *Hind*III sites are shown in the sequence including exon 1 (sequence from Jen et al, 1994 and AC000049). Regions 1, 2 and 3 of *p15*, analysed for their methylation status using a PCR-based assay, are shown at the top. The probe used for methylation analysis by Southern blotting (Herman et al, 1996) is shown at the bottom, along with the predicted sizes of restriction fragments for deletions of exon 1 of *p16*. All 5 tumours with homozygous *p15* deletions also had homozygous deletions of *p16* (data not shown).

### Lack of methylation of the CpG island in exon 1 of the *p15* gene in bladder tumours

The 5′ region of the *p15* gene contains a CpG island located around the transcription start site. It is therefore a good candidate for hypermethylation-associated inactivation (Herman et al, 1996). A methylation-sensitive restriction map of approximately 600-bp extending from the promoter region through exon 1 of the *p15* gene is shown in Figure 3. A PCR-based assay was used to analyse the methylation status of this CpG island. Double digestion with a restriction enzyme cutting the flanking regions (*Afl*II) and methylation-sensitive enzymes (*Cfo*I, *Hpa*II, *Eag*I, *Sac*II) was followed by amplification of regions 1, 2 and 3 (Figure 3). Normal urothelium showed no methylation in any of the three regions tested (Figure 4 and data not shown). Eight of the nine primary TCCs expressing *p15* that were tested were also unmethylated. Only one *p15*-expressing tumour was methylated in region 3 of exon 1, heavily at a *Sac*II and at a lower level at *Hpa*II restriction sites (Figure 4). After excluding the possibility of homozygous deletions of *p15*, the methylation status of five of the seven tumours that did not express *p15* was assessed. The five available samples showed no detectable methylation in any of the three regions tested. Restriction of genomic DNA from one normal urothelium and 10 tumours with the flanking enzyme *Hind*III, plus the methylation-sensitive enzyme *Eag*I, and Southern blotting with a *p15* exon 1 probe, confirmed the results obtained by the PCR-based assay (data not shown). The *p15* gene was unmethylated at this *Eag*I site in normal urothelium. All tumour tissues, both expressing and not expressing *p15*, were unmethylated at this site.

### mRNA levels and homozygous deletions of *p15* in bladder tumour cell lines

Of the 13 human bladder cell lines for which mRNA levels were analysed, 6 cell lines had no detectable *p15* mRNA and one cell line (EJ138) had low levels of *p15* mRNA (Table 3). In contrast with the tumours, all 6 bladder cell lines with no *p15* expression presented homozygous deletions of *p15*, always deleted with *p16* (data not shown). As expected, in the seven remaining tumour cell lines, which expressed *p15*, no homozygous deletions were detected. In the EJ138 cell line, which had very weak *p15* expression, the methylation status of *p15* exon 1 was studied by PCR and Southern blotting, and no abnormal DNA methylation was detected (data not shown).

### DISCUSSION

A critical area of chromosomal deletion at region 9p21–22 has been implicated in the genesis of various types of primary tumours, including bladder carcinomas. Three negative cell cycle regulators, p15, p16 and $p14^{ARF}$, encoded by two genes located in tandem in this region, have been identified as potential tumour suppressors. The role of p15 in carcinomas is unclear. Indeed, no intragenic mutation in *p15* has been reported and homozygous

**Table 3** Homozygous deletions of *p15* in bladder tumour cell lines according to *p15* mRNA levels. *p15* mRNA levels are the ratio of *p15* mRNA to *TBP* mRNA in semi-quantitative RT-PCR. The presence (+) or absence (–) of a homozygous deletion of the *p15* gene was determined as described in Materials and Methods

| Cell line | *p15* mRNA levels | Homozygous deletions of *p15* |
|---|---|---|
| HCV29 | 0 | + |
| RT112 | 0 | + |
| RT4 | 0 | + |
| UM-UC-3 | 0 | + |
| VM-CUB-1 | 0 | + |
| VM-CUB-3 | 0 | + |
| EJ138 | 0.1 | – |
| J82 | 0.4 | – |
| TCCSUP | 0.6 | – |
| HT1376 | 0.7 | – |
| T24 | 0.9 | – |
| 647V | 2.8 | – |
| JON53 | 3.9 | – |

© 2001 Cancer Research Campaign 

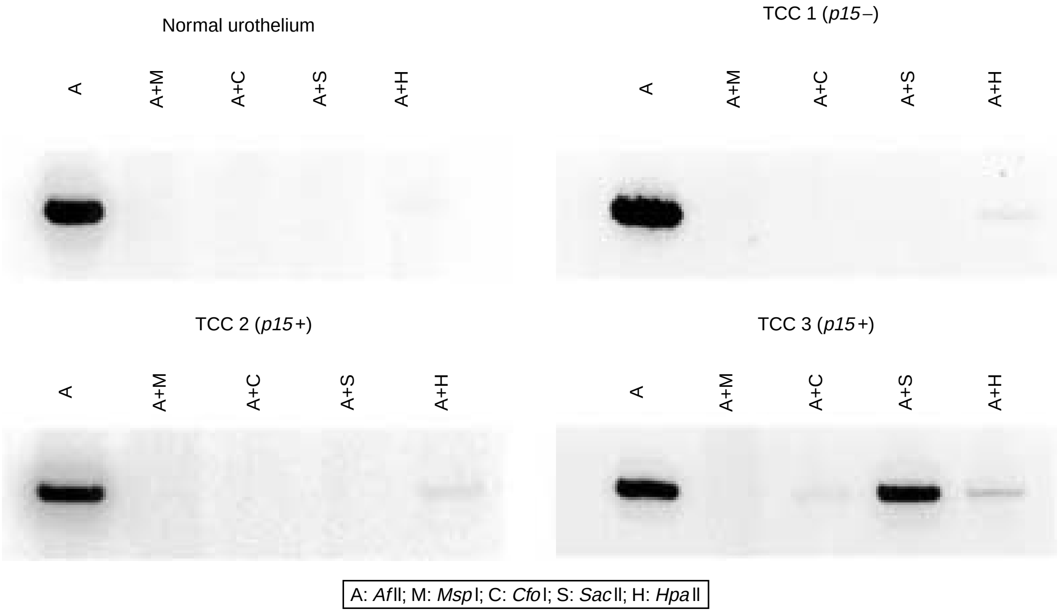

**Figure 4** Methylation status of the first exon of the *p15* gene. A representative methylation-sensitive PCR analysis (region 3 of exon 1) of normal urothelium and neoplastic cells from 3 TCCs is shown. *p15* + and *p15* are samples with or without *p15* expression respectively. The lanes are: PCR products obtained from DNA samples digested with *Afl*II (A : cutting outside the region analysed) as positive control; *Afl*II plus *Msp*I (A+M : cutting within the region analysed and methylation-insensitive) as negative control; *Afl*II plus *Cfo*I (A+C) or *Sac*II (A+S) or *Hpa*II (A+H) (all 3 cutting within the region analysed and methylation-sensitive)

deletions of *p15* almost always include the neighbouring *INK4*a/*ARF* locus. We investigated the possible involvement of *p15* in bladder carcinomas, by comparing *p15* mRNA levels in normal urothelium and a series of derived carcinomas.

We found that *p15* was expressed in the normal urothelium and that *p15* mRNA levels were significantly decreased in most superficial TCCs (66%). This decreased expression was not due to contamination of the tumour samples by normal tissue and that for several reasons: all the TCC samples used in this analysis were primarily composed of tumour cells, as assessed by histological examination; contamination of the tumour by the underlying compartments, the lamina propria and the smooth muscle, which express p15 at a level similar to that in urothelium, would have masked a decrease in *p15* mRNA level. Furthermore, the analysis of *p15* mRNA expression according to proliferation in normal urothelia showed that *p15* mRNA levels did not decrease in proliferative urothelia thus demonstrating that lower *p15* mRNA levels encountered in Ta-T1 tumours were in fact a tumour specific alteration and did not reflect a proliferative state.

Considering the known involvement of *p16* in the genesis of various tumours and because *p16* was located nearby *p15*, we have also investigated *p16* mRNA expression in those tumours. We found that *p16* mRNA levels were non-detectable in any of the normal bladder samples and increased with stage and grade of the TCCs. The absence of *p16* expression in normal bladder urothelium was in agreement with several other studies that did not find any *p16* expression in normal urothelium cultured or uncultured neither by RT-PCR nor Western blotting nor immunohistochemistry (Yeager et al, 1995; Stadler et al, 1996; Benedict et al, 1999). In TCCs, we have found that *p16* mRNA expression was not correlated to *p15* mRNA levels (correlation coefficient $R^2 = 0.26$) thus indicating that the expression of the two neighbouring *p15* and *p16* genes encoding related CDK inhibitors was differently regulated in normal and tumour tissues.

We investigated several possible explanations for *p15* mRNA down-regulation. Although loss of heterozygosity on chromosome band 9p21 is a common event, *p15* mRNA down-regulation in this case cannot be due to the loss of one copy of the *p15* gene, as in 26 of the 34 TCCs with decreased *p15* mRNA levels there was almost no *p15* mRNA. As the *p15* and *p16* loci are targets for homozygous deletions in bladder carcinomas, the loss of two copies of the *p15* gene may account for the down-regulation of *p15* reported herein. To avoid underestimation of homozygous deletions due to the presence of even small amounts of contaminating normal tissue, a semi-quantiative PCR assay was performed with a limited number of PCR cycles (see Materials and Methods). The frequency of homozygous deletions at the *p15* locus in primary TCCs in this study (18%) was similar to that in several other studies (Spruck et al, 1994; Orlow et al, 1995; Packenham et al, 1995; Williamson et al, 1995). The *p15* homozygous deletions that we observed were always associated with *p16* deletions and were present in less than half the primary bladder tumours with little or no *p15* expression. In primary tumours, homozygous deletions were not the sole mechanism of *p15* down-regulation. In contrast, the frequency of homozygous *p15* deletions was much higher in the bladder cell lines tested (about 50%) than in primary tumours. These deletions, in all but one case, account for the lack of expression in cell lines. Differences in the frequency of homozygous deletions in primary tumours and cell lines for certain tumour types including bladder carcinomas have been reported independently by many studies (Spruck et al, 1994; Southgate et al, 1995; Williamson et al, 1995). Homozygous deletions in cell lines may confer a long-term growth advantage in vitro (Spruck et al, 1994). Transcriptional repression by DNA methylation of the CpG island in the 5’ region of *p15* may be an additional mechanism of inactivation of this gene in primary bladder carcinomas. This mechanism, specifically involving the *p15* gene, has already been reported by Herman et al (1996) in leukaemias and some gliomas. In bladder tumours, and similarly to Gonzalez-Zulueta et al (1995), we observed no hypermethylation-associated inactivation of *p15* suggesting that this epigenetic mechanism is not involved in bladder carcinomas.

 *© 2001 Cancer Research Campaign*

The decreased *p15* expression in tumours without homozygous deletions, may be due to a pathway involved in *p15* regulation. As the p15 protein is a major mediator of the antiproliferative effects of TGF-β (Hannon and Beach, 1994), an abnormality in the TGF-β signalling pathway associated with the resistance of cancer cells to TGF-β-induced growth inhibition (Markowitz et al, 1996) could result in *p15* mRNA down-regulation.

It is interesting to note that *p15* mRNA levels were significantly decreased in most superficial TCCs, whereas 76% of invasive TCCs had *p15* mRNA levels similar to or higher than those found in normal urothelium. P15 and p16 are cell cycle regulatory proteins that prevent CDK4 activation. The cyclinD-CDK4 complex catalyses the phosphorylation of pRb, which releases E2F, resulting in G1 to S cell cycle progression. It has been reported that tumour cells with mutations in *Rb* express very high levels of wild-type p16 whereas pRb-positive tumour cells frequently show little or no p16 (Yeager et al, 1995; Hall and Peters, 1996). Similarly, we can suppose that a target molecule downstream from p15 may be inactivated in some invasive tumours, thereby inducing p15 up-regulation.

In conclusion, our results suggest that *p15* mRNA down-regulation is a frequent event in early neoplastic transformation of the urothelium and provide the first evidence for the possible involvement of this gene in carcinomas.

### ACKNOWLEDGEMENTS

We would like to thank Dr Christian Larsen for the critical review of the manuscript and Dr Jennifer Southgate for kindly providing cell lysates. This work was supported by the Association Claude Bernard, Université Paris XII, CNRS, Ligue Contre le Cancer (Comité de Paris and Comité du Val de Marne), Délégation à la Recherche Clinique (PHRC, AOA94015), the GEFLUC. D. Cappellen was awarded a fellowship from ARC and S. Gil Diez de Medina from the Ligue Contre le Cancer-Comité du Val de Marne.

### REFERENCES

Benedict WF, Lerner SP, Zhou J, Shen X, Tokunaga H and Czerniak B (1999) Level of retinoblastoma protein expression correlates with p16 (MTS-1/INK4A/CDKN2) status in bladder cancer. *Oncogene* **18**: 1197–1203

Cappellen D, Gil Diez de Medina S, Chopin D, Thiery JP and Radvanyi F (1997) Frequent loss of heterozygosity on chromosome 10q in muscle-invasive transitional cell carcinomas of the bladder. *Oncogene* **14**: 3059–3066

Coombs LM, Pigott D, Proctor A, Eydmann M, Denner J and Knowles MA (1990) Simultaneous isolation of DNA, RNA and antigenic protein exhibiting kinase activity from small tumor samples using guanidine isothiocyanate. *Anal Biochem* **188**: 338–343

de Boer WI, Rebel JMJ, Vermey M, de Jong AAW and van der Kwast TH (1994) Characterization of distinct functions for growth factors in murine transitional epithelial cells primary organotypic culture. *Exp Cell Res* **214**: 510–518

de Boer WI, Vermeij M, Gil Diez de Medina S, Bindels E, Radvanyi F, van der Kwast T and Chopin D (1996) Functions of fibroblast and transforming growth factors in primary organoid-like cultures of normal human urothelium. *Lab Invest* **75**: 147–156

Gil Diez de Medina S, Chopin D, El Marjou A, Delouvee A, Larochelle WJ, Hoznek A, Abbou C, Aaronson SA, Thiery JP and Radvanyi F (1997) Decreased expression of keratinocyte growth factor receptor in a subset of human transitional cell bladder carcinomas. *Oncogene* **14**: 323–330

Gil Diez de Medina S, Popov Z, Chopin DK, Southgate J, Tucker GC, Delouvee A, Thiery JP and Radvanyi F (1999) Relationship between E-cadherin and fibroblast growth factor receptor 2b expression in bladder carcinomas. *Oncogene* **18**: 5722–5726

Gonzalez-Zulueta M, Bender CM, Yang AS, Nguyen T, Beart RW, Van Tornout JM and Jones PA (1995) Methylation of the 5'CpG island of the p16/CDKN2 tumor suppressor gene in normal and transformed human tissues correlates with gene silencing. *Cancer Res* **55**: 4531–4535

Hall M and Peters G (1996) Genetic alterations of cyclins, cyclin-dependent kinases, and cdk inhibitors in human cancer. *Adv Cancer Res* **68**: 67–108

Hannon GJ and Beach D (1994) p15$^{INK4B}$ is a potential effector of TGF-β-induced cell cycle arrest. *Nature* **371**: 257–261

Herman JG, Merlo A, Mao L, Lapidus RG, Issa JPJ, Davidson NE, Sidranski D and Baylin SB (1995) Inactivation of the CDKN2/p16/MTS1 gene is frequently associated with aberrant DNA methylation in all common human cancers. *Cancer Res* **55**: 4525–4530

Herman JG, Jen J, Merlo A and Baylin SB (1996) Hypermethylation-associated inactivation indicates a tumor suppressor role of p15$^{INK4B1}$. *Cancer Res* **56**: 722–727

Jen J, Harper JW, Bigner SH, Bigner DD, Papadopoulos N, Markowitz S, Willson JKV, Kinzler KW and Volgestein B (1994) Deletion of p16 and p15 genes in brain tumors. *Cancer Res* **54**: 6353–6358

Kamb A (1995) Cell-cycle regulators and cancer. *TIG* **11**: 136–140

Markowitz SD and Roberts AB (1996) Tumor suppressor activity of the TGF-β pathway in human cancers. *Cytokine & Growth Factor Reviews* **7**: 93–102

Merlo A, Herman JG, Mao L, Lee DJ, Gabrielson E, Burger PC, Baylin SB and Sidransky D (1995) 5'CpG island methylation is associated with transcriptional silencing of the tumour suppressor p16/CDKN2/MTS1 in human cancers. *Nature Medicine* **1**: 686–692

Miyamoto H, Kubota Y, Fujinami K, Dobashi Y, Kondo K, Yao M, Shuin T and Hosaka M (1995) Infrequent somatic mutations of the p16 and p15 genes in human bladder cancer: p16 mutations occur only in low-grade and superficial bladder cancers. *Oncol Res* **7**: 327–330

Orlow I, Lacombe L, Hannon GJ, Serrano M, Pellicer I, Dalbagni G, Reuter VE, Zhang, ZF, Beach D and Cordon-Cardo C (1995) Deletion of the p16 and p15 genes in human bladder tumors. *J Natl Cancer Inst* **87**: 1524–1529

Packenham JP, Taylor JA, Anna CH, White CM and Devereux TR (1995) Homozygous deletions but no sequence mutations in coding regions of p15 or p16 in human primary bladder tumors. *Mol Carcinogenesis* **14**: 147–151

Radvanyi F, Christgau S, Baekkeskov S, Jolicoeur C and Hanahan D (1993) Pancreatic beta cells cultured from individual preneoplastic foci in a multistage tumorigenesis pathway: a potentially general technique for isolating physiologically representative cell lines. *Mol Cell Biol* **13**: 4223–4232

Reznikoff CA, Belair CD, Yeager TR, Savelieva E, Blelloch RH, Puthenveettil JA and Cuthill S (1996) A molecular genetic model of human bladder cancer pathogenesis. *Semin Oncol* **23**: 571–584

Serrano M, Hannon GJ and Beach D (1993) A new regulatory motif in cell-cycle control causing specific inhibition of cyclin D/CDK4. *Nature* **366**: 704–707

Sharpless NE and DePinho RA (1999) The INK4A/ARF locus and its two gene products. *Curr Opin Genet Dev* **9**: 22–30

Sherr CJ (1996) Cancer cell cycles. *Science* **274**: 1672–1677

Sherr CJ (1998) Tumor surveillance via the ARF-p53 pathway. *Genes & Dev* **12**: 2984–2991

Singer-Sam J, Yang TP, Mori N, Tanguay RL, Le Bon JM, Flores JC and Riggs AD (1990) In: *Nucleic Acid Methylation*, Clawson GA, Willis DB, Weissbach A and Jones PA (eds) pp 285–289. A R Liss: New York

Southgate J, Proffitt J, Roberts P, Smith B and Selby P (1995) Loss of cyclin-dependent kinase inhibitor genes and chromosome 9 karyotypic abnormalities in human bladder cancer cell lines. *Br J Cancer* **72**: 1214–1218

Spruck III CH, Gonzalez-Zulueta M, Shibata A, Simoneau AR, Lin MF, Gonzales F, Tsai YC and Jones PA (1994) P16 gene in uncultured tumours. *Nature* **370**: 183–184

Stadler WM and Olopade OI (1996) The 9p21 region in bladder cancer cell lines: large homozygous deletions inactivate the CDKN2, CDKN2B and MTAP genes. *Urol Res* **24**: 239–244

Stone S, Dayananth P, Jiang P, Weaver-Feldhaus, JM, Tavtigian SV, Cannon-Albright L and Kamb A (1995) Genomic structure, expression and mutational analysis of the p15 (MTS2) gene. *Oncogene* **11**: 987–991

UICC-American Joint Committee on Cancer (1992) *Manual for staging of cancer*. 4th edition. Lippincott: Philadelphia

WHO (1973) *Histological typing of urinary bladder tumours*. World Health Organization: Geneva

Williamson MP, Elder PA, Shaw ME, Devlin J and Knowles MA (1995) P16 (CDKN2) is a major deletion target at 9p21 in bladder cancer. *Hum Mol Genet* **4**: 1569–1577

Yeager T, Stadler W, Belair C, Puthenveettil J, Olopade O and Reznikoff C (1995) Increased p16 levels correlate with pRb alterations in human urothelial cells. *Cancer Res* **55**: 493–497

*© 2001 Cancer Research Campaign* 

## References

[PDF_00636] Benedict W. F., Lerner S. P., Zhou J., Shen X., Tokunaga H., Czerniak B. (1999). Level of retinoblastoma protein expression correlates with p16 (MTS-1/INK4A/CDKN2) status in bladder cancer.. Oncogene.

[PDF_00639] Cappellen D., Gil Diez de Medina S., Chopin D., Thiery J. P., Radvanyi F. (1997). Frequent loss of heterozygosity on chromosome 10q in muscle-invasive transitional cell carcinomas of the bladder.. Oncogene.

[PDF_00643] Coombs L. M., Pigott D., Proctor A., Eydmann M., Denner J., Knowles M. A. (1990). Simultaneous isolation of DNA, RNA, and antigenic protein exhibiting kinase activity from small tumor samples using guanidine isothiocyanate.. Anal Biochem.

[PDF_00660] De Medina S. G., Popov Z., Chopin D. K., Southgate J., Tucker G. C., Delouvée A., Thiery J. P., Radvanyi F. (1999). Relationship between E-cadherin and fibroblast growth factor receptor 2b expression in bladder carcinomas.. Oncogene.

[PDF_00655] Diez de Medina S. G., Chopin D., El Marjou A., Delouvée A., LaRochelle W. J., Hoznek A., Abbou C., Aaronson S. A., Thiery J. P., Radvanyi F. (1997). Decreased expression of keratinocyte growth factor receptor in a subset of human transitional cell bladder carcinomas.. Oncogene.

[PDF_00663] Gonzalez-Zulueta M., Bender C. M., Yang A. S., Nguyen T., Beart R. W., Van Tornout J. M., Jones P. A. (1995). Methylation of the 5' CpG island of the p16/CDKN2 tumor suppressor gene in normal and transformed human tissues correlates with gene silencing.. Cancer Res.

[PDF_00667] Hall M., Peters G. (1996). Genetic alterations of cyclins, cyclin-dependent kinases, and Cdk inhibitors in human cancer.. Adv Cancer Res.

[PDF_00669] Hannon G. J., Beach D. (1994). p15INK4B is a potential effector of TGF-beta-induced cell cycle arrest.. Nature.

[PDF_00676] Herman J. G., Jen J., Merlo A., Baylin S. B. (1996). Hypermethylation-associated inactivation indicates a tumor suppressor role for p15INK4B.. Cancer Res.

[PDF_00672] Herman J. G., Merlo A., Mao L., Lapidus R. G., Issa J. P., Davidson N. E., Sidransky D., Baylin S. B. (1995). Inactivation of the CDKN2/p16/MTS1 gene is frequently associated with aberrant DNA methylation in all common human cancers.. Cancer Res.

[PDF_00680] Jen J., Harper J. W., Bigner S. H., Bigner D. D., Papadopoulos N., Markowitz S., Willson J. K., Kinzler K. W., Vogelstein B. (1994). Deletion of p16 and p15 genes in brain tumors.. Cancer Res.

[PDF_00683] Kamb A. (1995). Cell-cycle regulators and cancer.. Trends Genet.

[PDF_00684] Markowitz S. D., Roberts A. B. (1996). Tumor suppressor activity of the TGF-beta pathway in human cancers.. Cytokine Growth Factor Rev.

[PDF_00686] Merlo A., Herman J. G., Mao L., Lee D. J., Gabrielson E., Burger P. C., Baylin S. B., Sidransky D. (1995). 5' CpG island methylation is associated with transcriptional silencing of the tumour suppressor p16/CDKN2/MTS1 in human cancers.. Nat Med.

[PDF_00690] Miyamoto H., Kubota Y., Fujinami K., Dobashi Y., Kondo K., Yao M., Shuin T., Hosaka M. (1995). Infrequent somatic mutations of the p16 and p15 genes in human bladder cancer: p16 mutations occur only in low-grade and superficial bladder cancers.. Oncol Res.

[PDF_00694] Orlow I., Lacombe L., Hannon G. J., Serrano M., Pellicer I., Dalbagni G., Reuter V. E., Zhang Z. F., Beach D., Cordon-Cardo C. (1995). Deletion of the p16 and p15 genes in human bladder tumors.. J Natl Cancer Inst.

[PDF_00697] Packenham J. P., Taylor J. A., Anna C. H., White C. M., Devereux T. R. (1995). Homozygous deletions but no sequence mutations in coding regions of p15 or p16 in human primary bladder tumors.. Mol Carcinog.

[PDF_00700] Radvanyi F., Christgau S., Baekkeskov S., Jolicoeur C., Hanahan D. (1993). Pancreatic beta cells cultured from individual preneoplastic foci in a multistage tumorigenesis pathway: a potentially general technique for isolating physiologically representative cell lines.. Mol Cell Biol.

[PDF_00705] Reznikoff C. A., Belair C. D., Yeager T. R., Savelieva E., Blelloch R. H., Puthenveettil J. A., Cuthill S. (1996). A molecular genetic model of human bladder cancer pathogenesis.. Semin Oncol.

[PDF_00708] Serrano M., Hannon G. J., Beach D. (1993). A new regulatory motif in cell-cycle control causing specific inhibition of cyclin D/CDK4.. Nature.

[PDF_00710] Sharpless N. E., DePinho R. A. (1999). The INK4A/ARF locus and its two gene products.. Curr Opin Genet Dev.

[PDF_00712] Sherr C. J. (1996). Cancer cell cycles.. Science.

[PDF_00713] Sherr C. J. (1998). Tumor surveillance via the ARF-p53 pathway.. Genes Dev.

[PDF_00718] Southgate J., Proffitt J., Roberts P., Smith B., Selby P. (1995). Loss of cyclin-dependent kinase inhibitor genes and chromosome 9 karyotypic abnormalities in human bladder cancer cell lines.. Br J Cancer.

[PDF_00721] Spruck C. H., Gonzalez-Zulueta M., Shibata A., Simoneau A. R., Lin M. F., Gonzales F., Tsai Y. C., Jones P. A. (1994). p16 gene in uncultured tumours.. Nature.

[PDF_00723] Stadler W. M., Olopade O. I. (1996). The 9p21 region in bladder cancer cell lines: large homozygous deletion inactivate the CDKN2, CDKN2B and MTAP genes.. Urol Res.

[PDF_00727] Stone S., Dayananth P., Jiang P., Weaver-Feldhaus J. M., Tavtigian S. V., Cannon-Albright L., Kamb A. (1995). Genomic structure, expression and mutational analysis of the P15 (MTS2) gene.. Oncogene.

[PDF_00733] Williamson M. P., Elder P. A., Shaw M. E., Devlin J., Knowles M. A. (1995). p16 (CDKN2) is a major deletion target at 9p21 in bladder cancer.. Hum Mol Genet.

[PDF_00736] Yeager T., Stadler W., Belair C., Puthenveettil J., Olopade O., Reznikoff C. (1995). Increased p16 levels correlate with pRb alterations in human urothelial cells.. Cancer Res.

[PDF_00647] de Boer W. I., Rebel J. M., Vermey M., de Jong A. A., van der Kwast T. H. (1994). Characterization of distinct functions for growth factors in murine transitional epithelial cells in primary organotypic culture.. Exp Cell Res.

[PDF_00651] de Boer W. I., Vermeij M., Diez de Medina S. G., Bindels E., Radvanyi F., van der Kwast T., Chopin D. (1996). Functions of fibroblast and transforming growth factors in primary organoid-like cultures of normal human urothelium.. Lab Invest.

